# Effects of nitrogen application rate, nitrogen synergist and biochar on nitrous oxide emissions from vegetable field in south China

**DOI:** 10.1371/journal.pone.0175325

**Published:** 2017-04-18

**Authors:** Qiong Yi, Shuanghu Tang, Xiaolin Fan, Mu Zhang, Yuwan Pang, Xu Huang, Qiaoyi Huang

**Affiliations:** 1 Institute of Agricultural Resources and Environment, Guangdong Academy of Agricultural Sciences, Guangzhou, China; 2 Key Laboratory of Plant Nutrition and Fertilizer in South Region, Ministry of Agriculture, Guangzhou, China; 3 Guangdong Key Laboratory of Nutrient Cycling and Farmland Conservation, Guangzhou, China; 4 College of Agriculture, South China Agricultural University, Guangzhou, China; Tennessee State University, UNITED STATES

## Abstract

Globally, vegetable fields are the primary source of greenhouse gas emissions. A closed-chamber method together with gas chromatography was used to measure the fluxes of nitrous oxide (N_2_O) emissions in typical vegetable fields planted with four vegetables sequentially over time in the same field: endive, lettuce, cabbage and sweet corn. Results showed that N_2_O fluxes occurred in pulses with the N_2_O emission peak varying greatly among the crops. In addition, N_2_O emissions were linearly associated with the nitrogen (N) application rate (*r* = 0.8878, *n* = 16). Excessive fertilizer N application resulted in N loss through nitrous oxide gas emitted from the vegetable fields. Compared with a conventional fertilization (N2) treatment, the cumulative N_2_O emissions decreased significantly in the growing seasons of four plant species from an nitrogen synergist (a nitrification inhibitor, dicyandiamide and biochar treatments by 34.6% and 40.8%, respectively. However, the effects of biochar on reducing N_2_O emissions became more obvious than that of dicyandiamide over time. The yield-scaled N_2_O emissions in consecutive growing seasons for four species increased with an increase in the N fertilizer application rate, and with continuous application of N fertilizer. This was especially true for the high N fertilizer treatment that resulted in a risk of yield-scaled N_2_O emissions. Generally, the additions of dicyandiamide and biochar significantly decreased yield-scaled N_2_O-N emissions by an average of 45.9% and 45.7%, respectively, compared with N2 treatment from the consecutive four vegetable seasons. The results demonstrated that the addition of dicyandiamide or biochar in combination with application of a rational amount of N could provide the best strategy for the reduction of greenhouse gas emissions in vegetable field in south China.

## Introduction

As an important greenhouse gas, nitrous oxide (N_2_O) not only plays an important role in global warming, it also contributes greatly to ozone depletion. The observational data monitored by the World Meteorological Organization (WMO) showed that by 2012 the average concentration of N_2_O increased to 325.1 ppb, which was 1.2 times higher than that in 1750 [1]. Because the warming potential of N_2_O is 298 times that of CO_2_, N_2_O emissions have received more attention. Agriculture contributes about 58% of total anthropogenic N_2_O emissions, and soils serve as the main approach of these emissions [2]. Increase levels of atmospheric N_2_O contribute about 6% of the overall global warming effect, with almost 80% of N_2_O is emitted from agricultural lands; this N_2_O originates from N fertilizers, soil disturbance and animal waste [3]. Over the long term, agricultural N_2_O emissions are projected to increase by 35%-60% by 2030; this increase is projected to be caused by increases in application nitrogen (N) fertilizer and in animal manure production [4]. Therefore, effective mitigation measures used to mitigate N_2_O emission from soil without sacrificing crop yield are urgently needed.

About 20% of the China’s direct N_2_O emission in the 1990s came from vegetable fields [5]. Vegetable crops cover about 1.35 million hm^-2^ in Guangdong Province ranking it fourth in the entire country, The Pearl River Delta region serves as the main vegetable production area, accounting for 37.6% of the total vegetable growing area in Guangdong Province; this region produces 32.75 million tons of vegetables per year [6–7]. Vegetable fields, a land use type with highly intensive use as well as a high rate of nitrogen application and frequent irrigation, are one of the most abundant land cover types that contribute greatly to greenhouse gas emissions in China [8]. Leaching and N_x_O emission are the primary N loss pathways in vegetable fields, especially when high N application rates are used[9]. Surface soil N and environmental conditions are crucial for determining the short-term N_2_O discharge during topdressing in greenhouse vegetable cultivation [10]. To reduce greenhouse gas emissions and alleviate the pressure on global warming potential (GWP), scientists have shown great interest in reducing emissions of greenhouse gases in recent years. Optimizing fertilizer N rates and applying nitrification inhibitors or changing from NH_4_^+^ to NO_3_^-^ based fertilizers can serve as effective measures for reducing N_2_O emissions [11–12]. The addition of liming in soil with enriched fertilizer N could reduce N_2_O emission, because the reduction of N_2_O underground is an important process that limits N_2_O emissions [13]. A markedly lower GWP, greenhouse gas intensity (GHGI) and enhance yields were observed when using the nitrification inhibitor, nitrapyrin and biological nitrification inhibitor treatments when compared to urea and a nitrification inhibitor, dicyandiamide (DCD) treatments in vegetable ecosystems [14]. Another research showed that the combination of chemical N fertilizer and manure with biochar (BC) at 30 Mg hm^-2^ provided the most effective measures for reducing N_2_O emissions in vegetable production [15]. The addition of BC increased soil organic carbon and total N content, vegetable yield and net ecosystem economic budget although it resulted in reduced net GWP and GHGI [16–17].

The emission of N_2_O in vegetable fields is largely influenced by the cropping system used as well as by temperature, precipitation, fertilization, and vegetable species and so on. A simple short term comparison of vegetable greenhouse gas emissions among different cropping systems will provide inaccurate and unreasonable results. Although the dynamics of greenhouse gases emissions have been observed extensively in farmland, only very limited studies have been conducted related to technology that can be used to reduce greenhouse gases emissions using an evaluation index combined with N management in a vegetable field. Nevertheless, many studies have shown that DCD or BC are effective in reducing N_2_O emissions, although it remained unclear which of these two materials would provide better results. More studies should be conducted that using more appropriate evaluation criterion to analyze the distinction between DCD and BC. The seasonal dynamics of N_2_O fluxes were measured in a typical vegetable field planted with four vegetables grown and harvested consecutively over four growing seasons: endive, lettuce, cabbage and sweet corn. Our mainly hypotheses were that: (1) the seasonal dynamics emission fluxes and cumulative emission of N_2_O would be increased with the increase of nitrogen fertilizer in vegetable fields, (2) the N_2_O emission of DCD and BC treatment would be decreased when compared with conventional treatment, and (3) the yield-scaled N_2_O emission would be increased with the higher application of N fertilizer and the yield-scaled N_2_O emission of DCD and BC treatment would be reduced compared with conventional treatment. Yield-scaled N_2_O emission could be regard as an effective indicator to assess and balance the agricultural productivity with N_2_O emissions under this type of cultivation system.

## Materials and methods

### Description of the experiment

**Fig 1:**
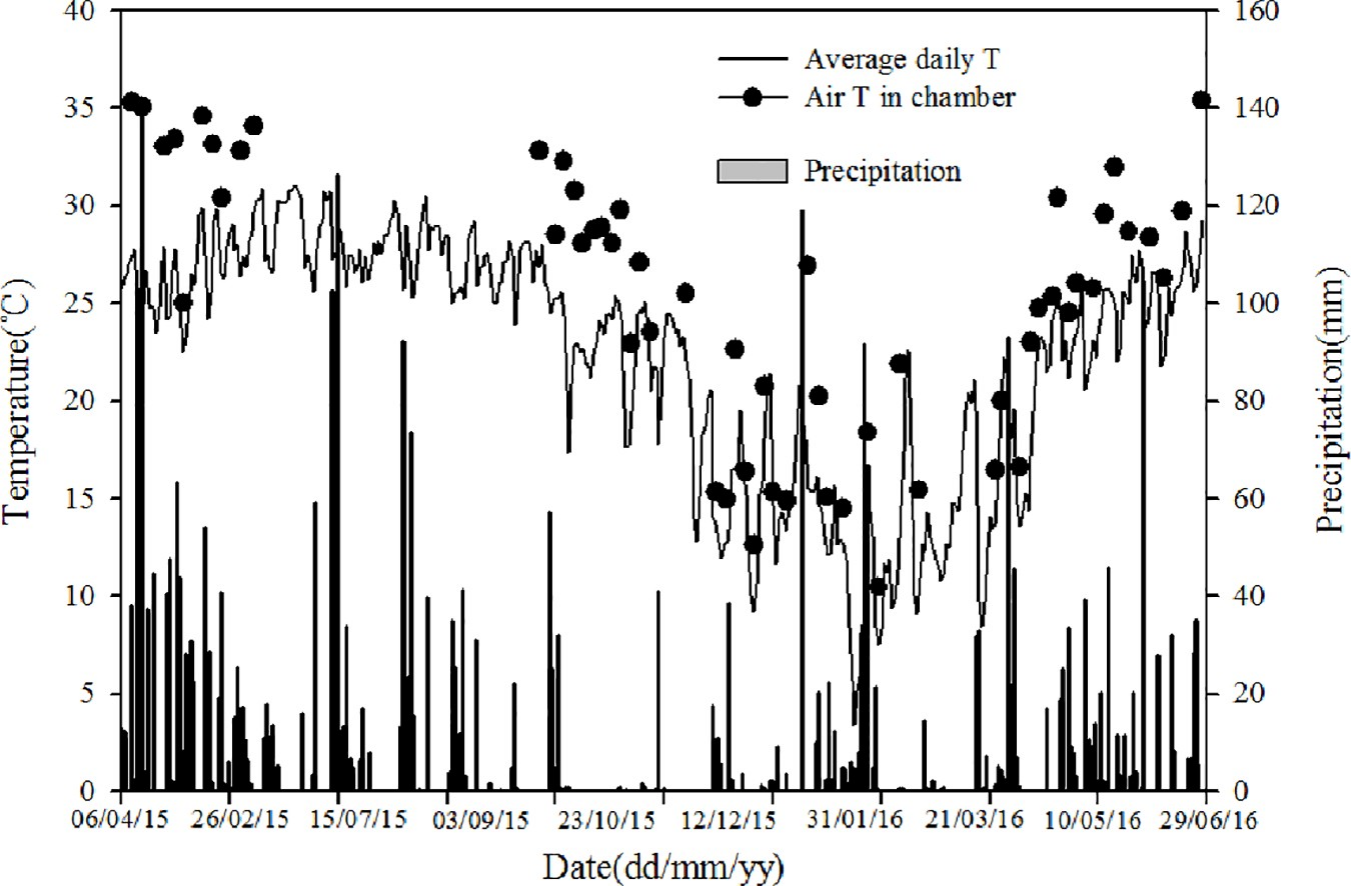
Air temperature inside and outside chamber and precipitation during consecutive four vegetable seasons.

A field experiment was conducted with four crops planted and harvested independently and consecutively from Apr. 2015 to Jun. 2016 (Table 1). The experiment field was located at the test base of Guangdong Academy of Agricultural Sciences (23°8′52′′N, 113°20′36′′E). The region experiences a typical subtropical maritime monsoon climate with an annual mean temperature and rainfall of 22.5°C and 1517 mm, respectively. About 73.8% of all precipitation is received from March to August. The air temperature and precipitation data were obtained from nearby weather station (Fig 1, S1 Fig).

**Table 1:** Cultivation time periods and N fertilization amounts and methods for four sequentially planted crops: endive, lettuce, cabbage, and sweet corn.

Vegetation	Growth period (dd/mm/yy)	Treatment (kg N ha^-1^)						Topdressing	Ration of basal N/dress N
		N0	N1	N2	N3	N2_DCD	N2_BC		
Endive	29/04/15-18/06/15	0	90	180	270	180	180	14/05/15, 01/06/15	0.3:(0.4:0.3)
Lettuce	29/09/15-16/11/15	0	75	150	225	150	150	12/10/15, 02/11/15	0.3:(0.4:0.3)
Cabbage	02/12/15-18/02/16	0	90	180	270	180	180	17/12/15, 04/01/16	0.3:(0.4:0.3)
Sweet corn	15/03/16-02/06/16	0	180	360	540	360	360	05/04/16, 11/04/16, 20/04/16	0.15:(0.3:0.15:0.4)

N0-no fertilizer N treatment; N1-low N application rate treatment (435 kg N ha^-1^); N2-conventional N application rate treatment (870 kg N ha^-1^); N3-high N application rate treatment (1305 kg N ha^-1^). N2_DCD- conventional N application rate treatment plus 5% of N fertilizer DCD, N2_BC- conventional N application rate treatment incorporated with 10 Mg ha^−1^ of BC.

Four consecutive vegetable crops, i.e., endive (*Cichorium endivia* L.), lettuce (*Lactuca sativa var*. *ramosa* Hort.), cabbage (*Brassica oleracea* L. *var*. *capitate* L.) and sweet corn (*Zea mays* L.) were cultivated from 29 April 2015 to 2 June 2016.The soil properties in the top 20 cm of the latosolic red soil at the site were as follows: pH 4.88, bulk density 1.36 g cm ^-3^, organic carbon 20.5 g kg^−1^, and total N 1.29 g kg^−1^. The experiment consisted of six treatments: (1) no fertilizer N treatment (N0), (2) low N application rate treatment with 435 kg N ha ^-1^ (N1), (3) conventional N application rate treatment with 870 kg N ha^-1^ (N2), (4) high N application rate treatment with 1305 kg N ha ^-1^ (N3), (5) N2 plus 5% of N fertilizer synergist (N2_DCD), (6) N2 incorporated with10 Mg ha^-1^of biochar (N2_BC). All the plots (each plot was 10 m^2^) were arranged in a completely randomized design with three replications. According to the local practice, urea (N 46%), superphosphate (P_2_O_5_ 12%) and potassium sulfate (K_2_O 50%) were used to maintain soil nutrient balance and crop growth. 75 kg P_2_O_5_ ha ^-1^ and 165 kg K_2_O ha ^-1^ were applied in the first three kinds of crops, although, 120 P_2_O_5_ ha^−1^ and 300 kg K_2_O ha^−1^ s were applied to sweet corn during the growing season. Phosphate fertilizer was applied as basal fertilization, and potash fertilizer was applied with nitrogen fertilizer in the same proportion. DCD was applied along with fertilizer N although BC was applied along with basal fertilization. The growth period of each crop type and the dominant N fertilization practices (including N application rate, time of topdressing and ratio of basal N to dress N) are shown in Table 1. According to the local watering methods, the frequency of irrigation at the early stage, especially after transplanting was relatively high. The timing and amount of irrigation was dependent on the weather conditions.

### Sampling and measurements

The closed-chamber method was used to determine the fluxes of N_2_O in each plot, and the concentrations of N_2_O were measured using an automated gas chromatograph (Agilent 7890B, USA) equipped with an electron capture detector (ECD). Gas samplings were conducted from 4 May 2015 to 28 June 2016 over 421 days. The gas collection device consisted of a chamber (0.4 m width × 0.4 m length × 0.4 m height) made of organic glass material with a stainless-steel base that was inserted into the ground. Generally, N_2_O flux was measured twice a week during the growing seasons. The sampling time for each chamber was 30 min in each treatment plot between 8:00 am and 12:00 am. Gas samples were collected using an injection syringe that was then taken to the laboratory as soon as possible to measure the concentration of N_2_O. Air temperatures outside and inside each sampling chamber were measured simultaneously with soil temperature and gravimetric moisture content at 5 cm depth for each treatment during the process of gas collection. The soil mineral nitrogen (NO_3_^−^-N and NH_4_^+^-N) samples collected at important growing stages or after fertilization were analyzed with Continuous Flow Analysis (FUTURA II, Alliance, France). The vegetable yields were calculated from the edible part of first three crops and aboveground biomass of sweet corn.

### Statistical analysis

N_2_O flux was calculated by using a temporal increase in N_2_O concentration in the chamber over time, and using Eq (1):

$$N_{2}O\;\mathrm{flux}\;\left(\mu g∙m^{-2}∙h^{-1}\right)=\rho \times \frac{V}{A}\times \frac{\mathrm{dc}}{\mathrm{dt}}\times \frac{273}{273+T}$$ (1)

where ρ is the density of N_2_O under standard state, $\frac{\mathrm{dc}}{\mathrm{dt}}$ is the change rate of N_2_O concentration along with time, *V* is the volume of the chamber, *A* is the cover area of the chamber and *T* is the air temperature in chamber during sampling.

The cumulative N_2_O emissions were calculated as the sum of daily estimates of N_2_O flux obtained by linear interpolation between two adjacent sampling dates, with an assumption that N_2_O flux measured on a sampling date was a representative of the average daily N_2_O emissions.

The water-filled pore space (WFPS) was calculated using Eq (2):

$$\mathrm{WFPS}\left(\%\right)=\left(\mathrm{volumetric}\;\mathrm{water}\;\mathrm{content}/\mathrm{total}\;\mathrm{soil}\;\mathrm{porosity}\right)\times 100$$ (2)

where total soil porosity = 1 − (soil bulk density/2.65), with 2.65 (g cm^−3^) being the assumed particle density of the soil.

Yield-scaled N_2_O emissions (g N_2_O-N kg^−1^ aboveground N uptake) were calculated using Eq (3) [18]:

$$\mathrm{Yield}\text{-}\mathrm{scaled}\;N_{2}O\;\mathrm{emission}=(\mathrm{Cumulative}\;N_{2}O\;\mathrm{emission}/\mathrm{aboveground}\;N\;\mathrm{uptake})$$ (3)

where aboveground N uptake denotes the total amount of N in aboveground biomass (kg N ha^−1^).

A general linear model (GLM) procedure was used for analysis of experimental data. Analysis of variance using Duncan’s new multiple at a 5% confidence level was performed on the N_2_O fluxes, the cumulative N_2_O emissions and yield-scaled N_2_O emissions. The correlation between N_2_O fluxes and the N application rate, mineral N content and N application rate was analyzed by a linear model procedure. All data were analyzed using the SAS software package for Windows (SAS 9.0).

## Results

### Dynamic changes of N_2_O fluxes and accumulation of N_2_O emissions under different N application rates

**Fig 2:**
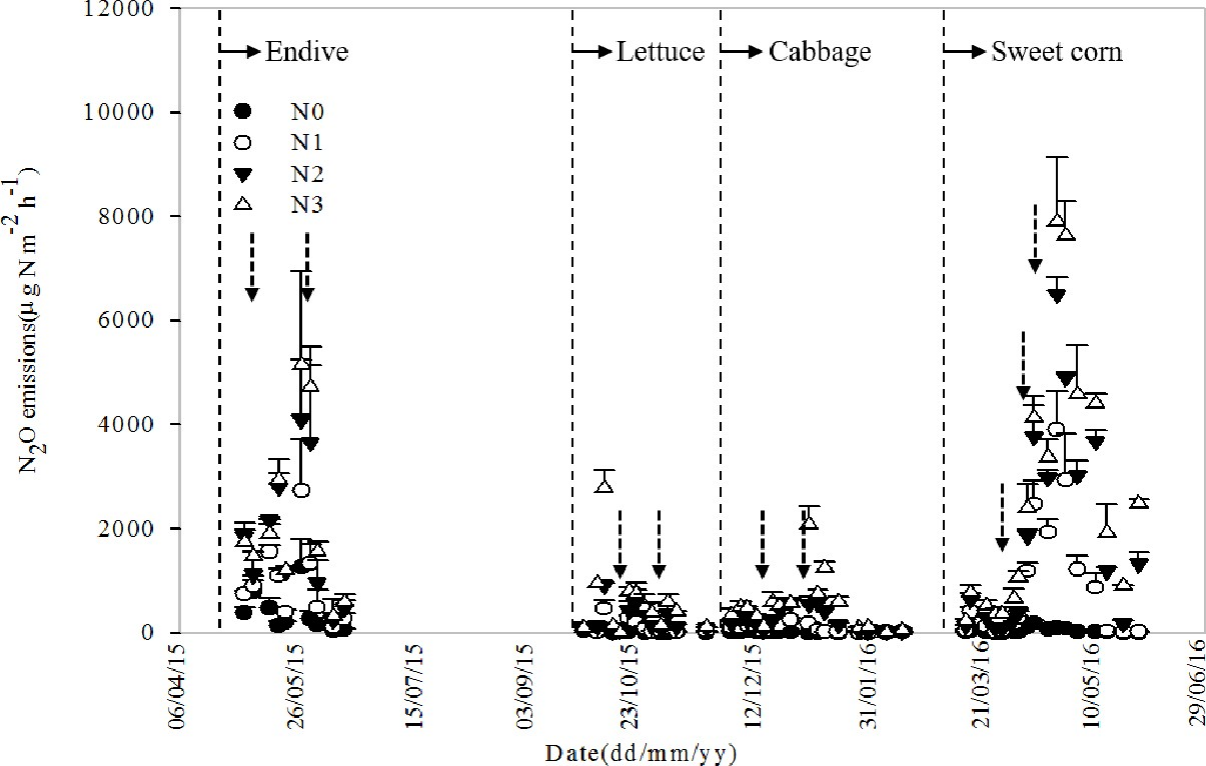
Temporal changes in N_2_O fluxes under different N application rates during four consecutive cropping seasons. The dotted lines in figure indicate transplanting/sowing of each crop, while the dashed arrows indicate fertilization time. N0-no fertilizer N treatment; N1-low N application rate treatment (435 kg N ha ^-1^); N2-conventional N application rate treatment (870 kg N ha ^-1^); N3-high N application rate treatment (1305 kg N ha ^-1^). The bars represent the standard error of the means (*n* = 3).

Dynamics changes were observed in N_2_O emissions fluxes during the growing seasons of the four crops (Fig 2, S2 Fig). The results showed that increases in N_2_O fluxes were closely related to the rate of N application. The N_2_O emissions occurred in pulses and the peak of N_2_O emissions varied greatly with crop. In addition, the peak value of N_2_O emissions increased with an increase in the N application rate. During the growth periods for endive, lettuce and sweet corn, N_2_O emissions peaked at 30 days after transplanting (DAT) for all treatments, 10 DAT and 41–45 days after sowing (DAS), respectively. However, in cabbage, the N_2_O emissions peaked at inconsistent times, a finding that may have been caused by the relatively low emission peak and low N_2_O concentration.

**Fig 3:**
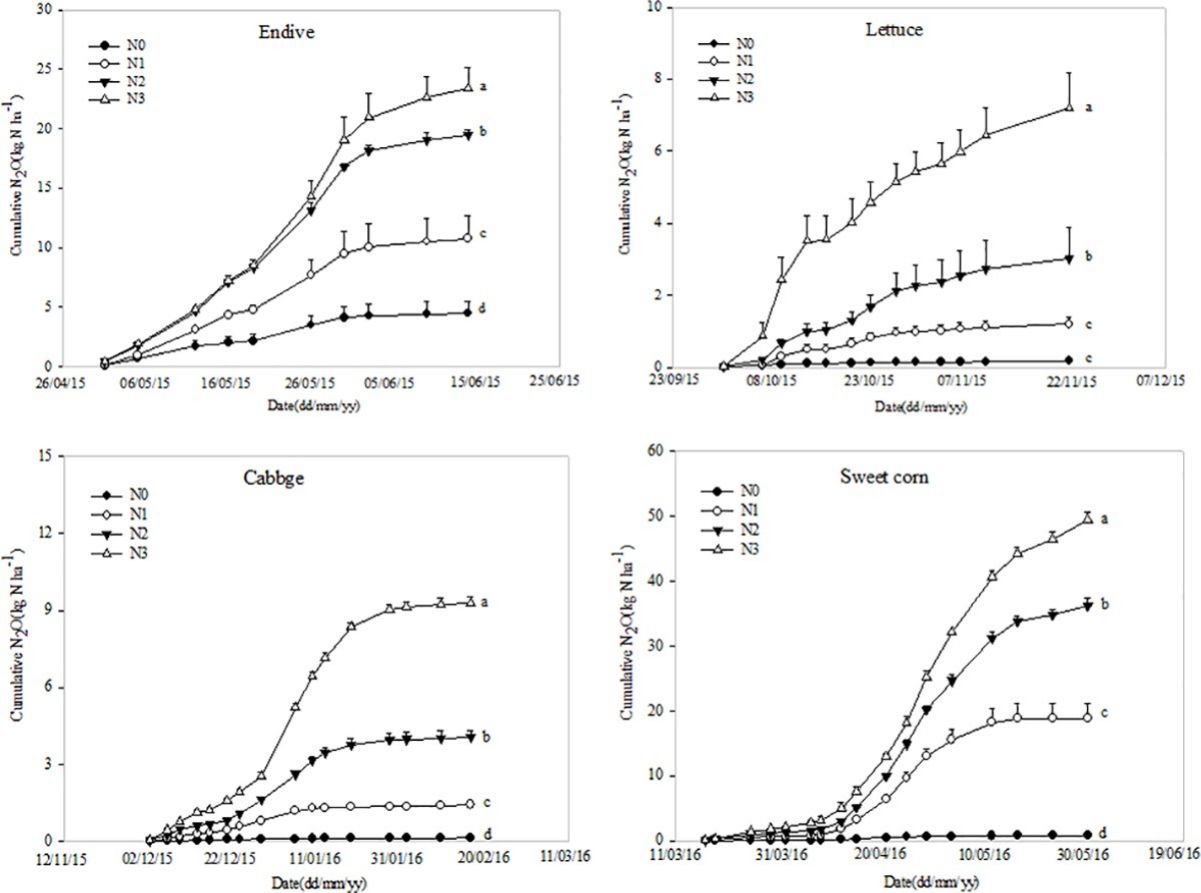
Cumulative N_2_O emissions from different N application rates in four consecutive cropping seasons. Different letters within each growing season indicated difference among treatments at *P*<0.05 level by Duncan’s new multiple range test. N0-no fertilizer N treatment; N1-low N application rate treatment (435 kg N ha^-1^); N2-conventional N application rate treatment (870 kg N ha^-1^); N3-high N application rate treatment (1305 kg N ha^-1^). The bars represent the standard error of the means (*n* = 3).

Cumulative N_2_O fluxes of treatments with different N application rates for each crop growing season fluctuated greatly from 5.6 to 89.7 kg N ha ^-1^ (Fig 3, S3 Fig). When the different crops were compared, the lettuce growing season showed the lowest cumulative N_2_O emissions among all N application treatments (except for the N0 treatment), accounting for less than 8.0% of total emissions from the observation periods. In contrast, the highest cumulative N_2_O emission occurred during the sweet corn growing season, accounted for more than 46.1% of the total emissions (except for N0 treatment) during all four cropping seasons. This result was not only caused by the high level of N fertilization, but could also be partially attributed to the change in fertilization method to furrow application of the base fertilizer in sweet corn. Clearly, the trends cumulative N_2_O emissions among different treatments during the growing seasons of four species were almost similar. A significant difference in cumulative N_2_O emissions was observed between different N application levels in each growing season (N3 > N2 > N1 > N0), which indicated that N_2_O emissions in vegetable fields are strongly affected by fertilizer N input. Great N application input resulted in more N_2_O emission.

### Dynamic seasonal emission fluxes and cumulative emission characteristics of N_2_O between DCD and BC

**Fig 4:**
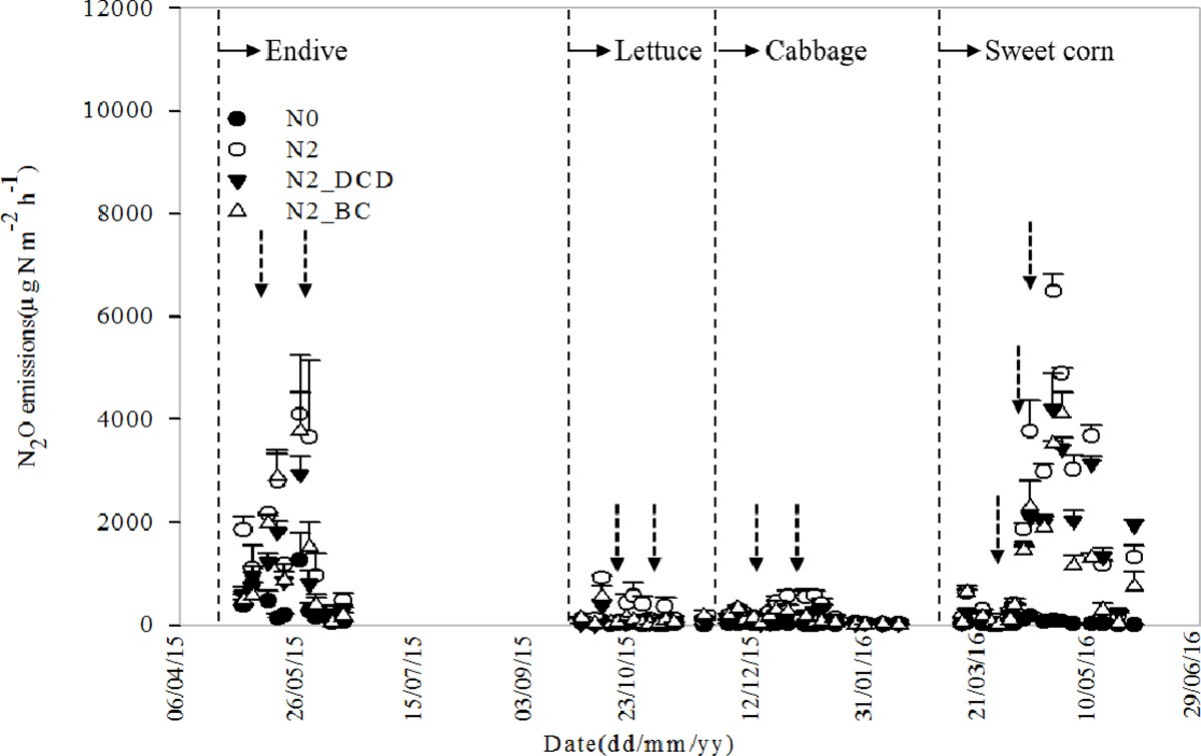
Temporal changes in N_2_O fluxes between dicyandiamide (DCD) and biochar (BC) during four consecutive cropping seasons. The dotted lines in figure mean transplanting/sowing of each crop, although the dashed arrows mean fertilization incident. N0-no fertilizer N treatment; N2-conventional N application rate treatment (870 kg N ha^-1^); N2_DCD-conventional N application rate treatment plus 5% of DCD, N2_BC-conventional N application rate plus incorporated with 10 Mg ha^−1^ of BC.

The addition of DCD and BC could effectively reduce seasonal N_2_O emission fluxes when compared with the N2 treatment (Fig 4, S4 Fig). N_2_O emission peaks of N2_DCD and N2_BC from four crop growing seasons were lower than that of N2 treatment.

**Fig 5:**
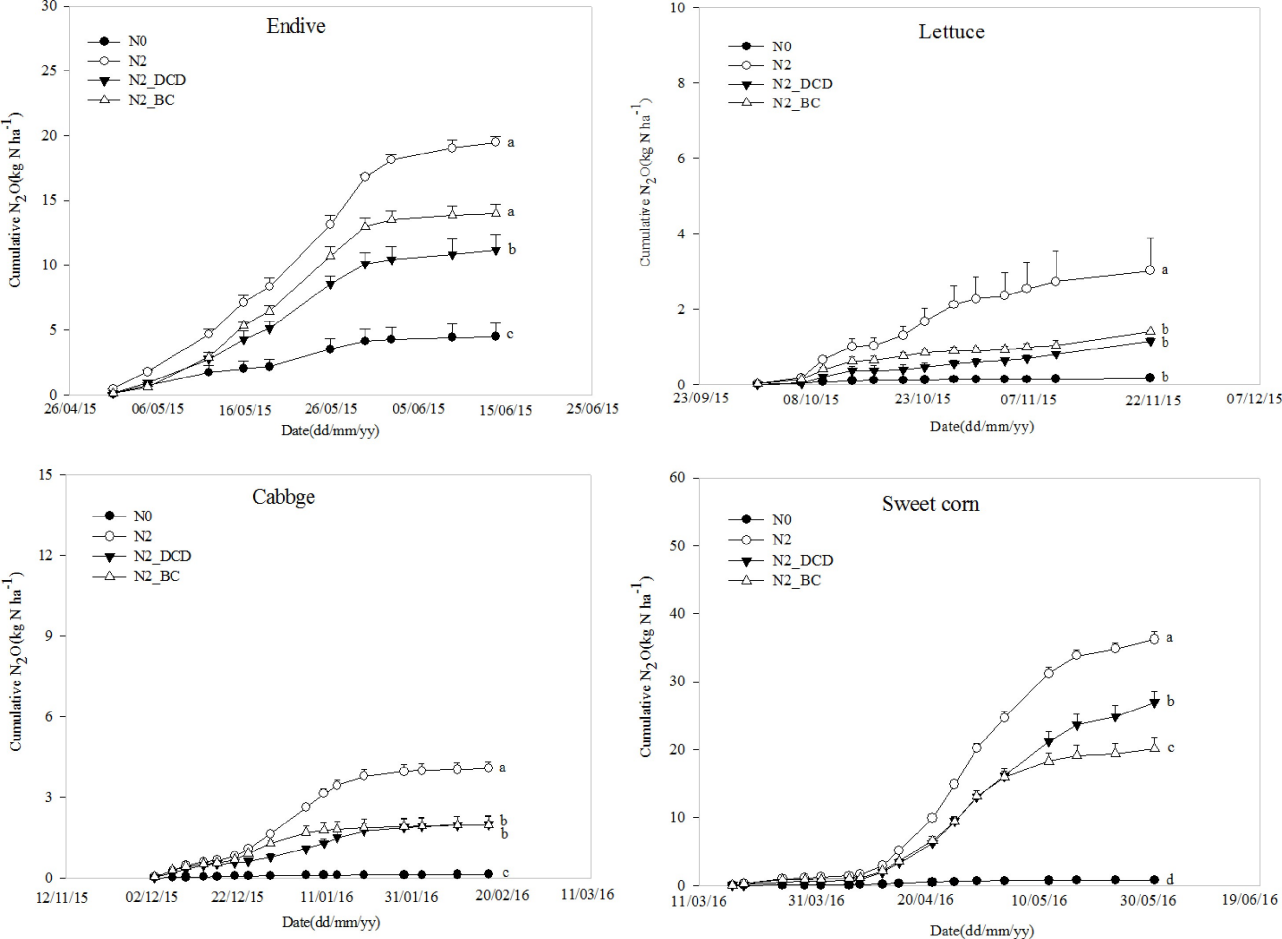
Cumulative N_2_O emission characteristics between dicyandiamide (DCD) and biochar(BC) treatment in four consecutive cropping seasons. N0-no fertilizer N treatment; N2- conventional N application rate treatment (870 kg N ha^-1^); N2_DCD-conventional N application rate treatment plus 5% of DCD, N2_BC-conventional N application rate plus incorporated with 10 Mg ha^−1^ of BC.

Overall, when compared with the treatment using the same amount of N fertilizer, the N2_DCD and N2_BC treatments significantly decreased cumulative N_2_O emissions in the observation periods by 34.6% and 40.8%, respectively. The cumulative data showed that compared with the N2 treatment, cumulative N_2_O emissions from the N2_DCD treatment decreased by 42.8%, 61.8%, 54.0% and 25.7% in the endive, lettuce, cabbage and sweet corn growing seasons, respectively. Meanwhile, the N2_BC treatment resulted in decreased N_2_O emissions by 28.4%, 53.6%, 56.9% and 44.5% in comparison with the N2 treatment for the same four crop growing seasons. Interestingly, the effects of N2_DCD and N2_BC treatments on cumulative N_2_O were quite different during the crop seasons of four crops grown consecutively (Fig 5, S5 Fig). In the endive season, the N2_DCD treatment resulted in significantly reduced cumulative N_2_O emissions when compared with the N2_BC treatment. In the lettuce and cabbage seasons, no significant differences were observed between N2_DCD and N2_BC on the reduction of the cumulative N_2_O emissions. Until sweet corn season, the BC treatment resulted in significantly reduced cumulative N_2_O emissions when compared with the N2_DCD treatment. The results indicated that the effects of BC on N_2_O emission reduction became more and more obvious over time.

### Effects of N application rate on yield-scaled N_2_O emissions

**Fig 6:**
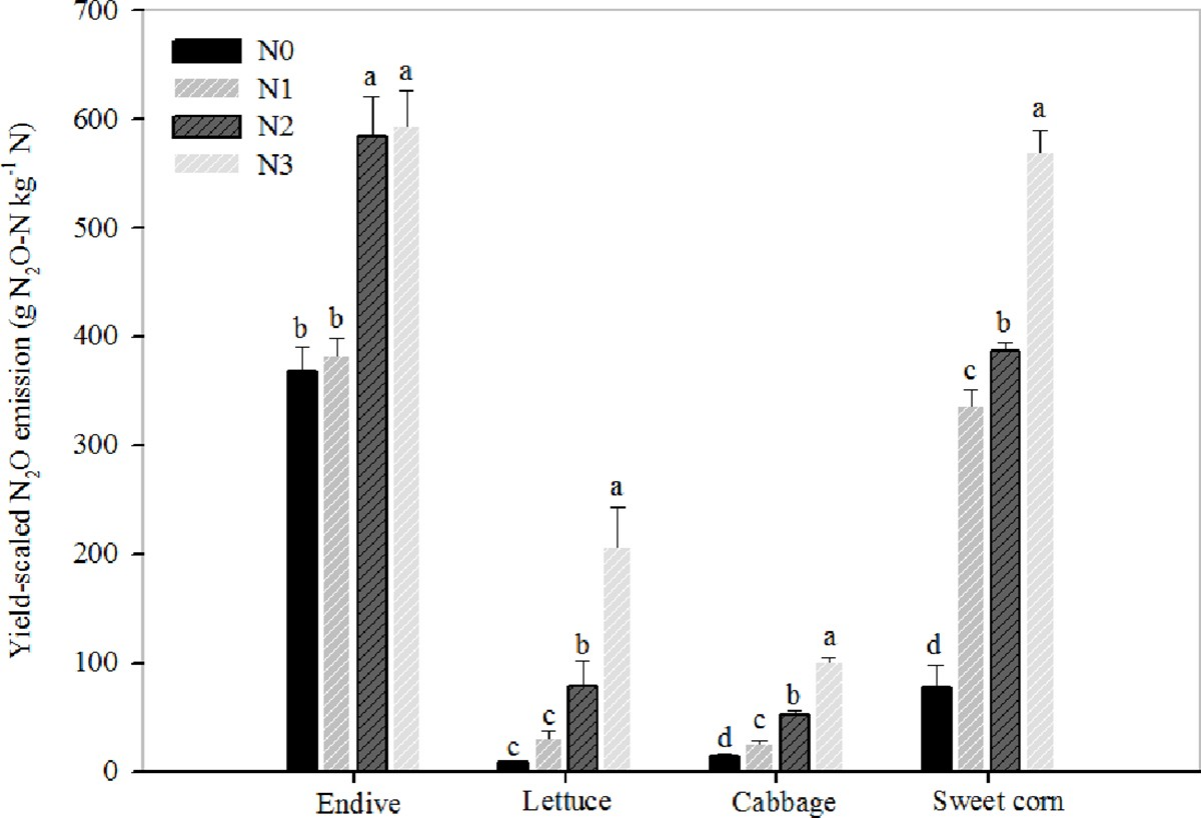
Effects of different N application rate on yield-scaled N_2_O emissions from four consecutive cropping seasons. N0-no fertilizer N treatment; N1, low N application rate treatment (435 kg N ha^-1^); N2-conventional N application rate treatment (870 kg N ha^-1^); N3-high N application rate treatment (1305 kg N ha^-1^). The yield here refers to edible part of a vegetable in first three crops and aboveground biomass of sweet corn. Different letters indicate significantly difference between treatments at *P*<0.05 by Duncan’s new multiple range test.

The yield-scaled N_2_O emissions from different N application rates varied greatly with crops and N applications ranging from 8.2 to 593.6 g N_2_O-N kg^−1^ N (Fig 6, S6 Fig). The yield-scaled N_2_O emissions in endive and sweet corn seasons were relatively higher than those of lettuce and cabbage seasons, which had the same trend of cumulative N_2_O emissions. This may have occurred because of the variations of climate in different seasons and the differences of the production index. The yield-scaled N_2_O emissions in four consecutive growing seasons increased with an increase in the N application rate (Fig 6, S6 Fig). No significant difference in yield-scaled N_2_O emissions was observed between the N0 and N1 treatments in the first two vegetable growing seasons. However, a significant difference was observed in yield-scaled N_2_O emissions between N0 and N1 in the last two vegetable growing seasons. The N3 treatment resulted in significantly increased yield-scaled N_2_O emissions by 1.7%, 163.6%, 93.5% and 47.4% when compared with the N2 treatments. The results indicated that the continuous application of N fertilizer, especially for the high N fertilizer treatment, resulted in the risk of yield-scaled N_2_O emissions.

### Effects of DCD and BC on yield-scaled N_2_O emissions

**Fig 7:**
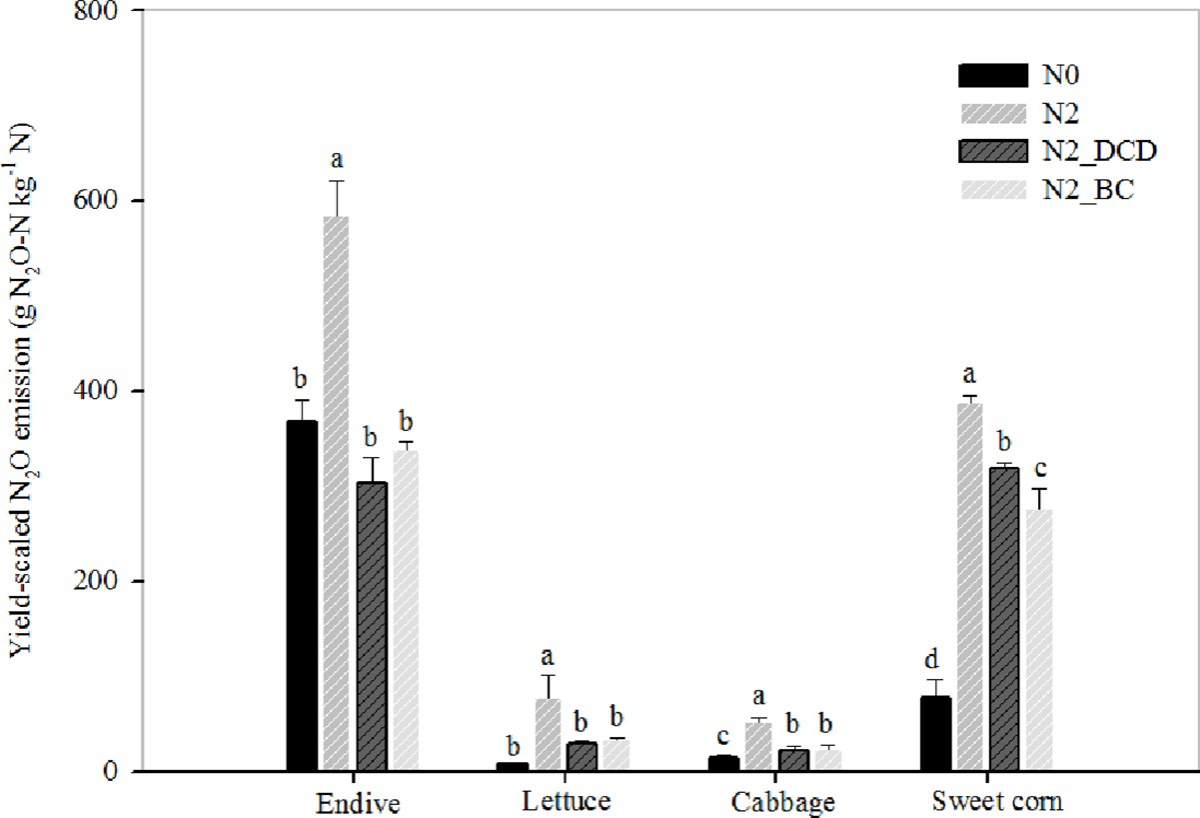
Effects of dicyandiamide (DCD) and biochar (BC) on yield-scaled N_2_O emissions from four consecutive cropping seasons. N0-no fertilizer N treatment; N2-conventional N application rate treatment (870 kg N ha^-1^); N2_DCD-conventional N application rate treatment plus 5% of DCD, N2_BC-conventional N application rate plus incorporated with 10 Mg ha^−1^ of BC. The yield here refers to edible part of a vegetable in first three crops and aboveground biomass of sweet corn. Different letters indicate significantly difference between treatments at P<0.05 by Duncan’s new multiple range test.

The yield-scaled N_2_O emissions from DCD and BC also varied greatly among crops ranging from 8.7 to 583.9 g N_2_O-N kg^−1^ N (Fig 7, S7 Fig). Compared with the N2 treatment, the N2_DCD treatment resulted in significantly decreased the yield-scaled N_2_O emissions by 48.1%, 61.4%, 56.5% and 17.6% in endive, lettuce, cabbage and sweet corn, respectively. Similarly, the application of the BC treatment also resulted in significantly reduced the yield-scaled N_2_O emissions by 42.2%, 56.8%, 55.0% and 28.7% in the same four crops, respectively. On average, the DCD and BC treatments resulted in decreased yield-scaled N_2_O-N emissions by 45.9% and 45.7% when compared with the N2 treatment, which indicated that the effects of the nitrification inhibitor (DCD) and BC on N_2_O emission reduction under the conditions of this experiment were quite remarkable, and the effects of BC on N_2_O emission reduction was better than DCD to some extent, particularly in the late crops of the test.

## Discussions

### The influence of different N fertilizer levels on N_2_O emissions

**Fig 8:**
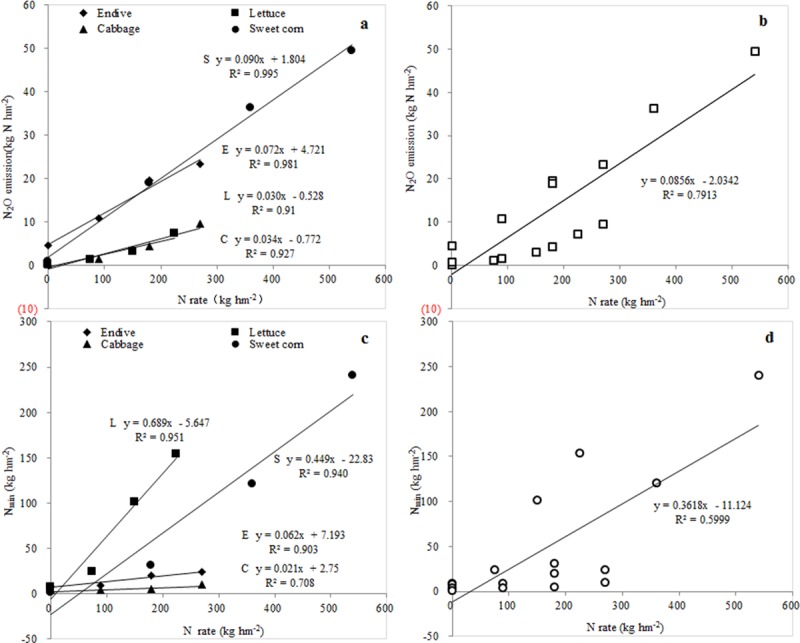
The correlation between N_2_O emissions, mineral N content and N application rate from vegetable fields. E-Endive, L-Lettuce, C-Cabbage and S-Sweet corn. N_min_-soil mineral nitrogen, the sum of NO_3_^−^-N and NH_4_^+^-N. a and b indicate a significant relationship exists between N application rate and N_2_O emissions in each growing season and in four consecutive seasons, respectively, c and d mean a significant correlation exists between N_2_O emissions and N_min_ in each growing season and in four consecutive seasons, respectively.

Nitrogen fertilization markedly influenced the soil N_2_O emission, although the effects of N fertilization were quite different in terms of nitrogen applications rates and types, crops, and seasons [19]. Both fertilization and plant types significantly altered N_2_O emission [20]. Small changes in N fertilizer can have a substantial environmental impact. A change from 75 to 50 kg N hm^-2^ reduced the GWP per hm^-2^ by 18% [21]. It is usually assumed that N_2_O emissions will increase with an increase in the N application rate [22]. Conversely, some studies have reported that there was a nonlinear response of N_2_O emission to incremental additions of N fertilizer [23], and the N_2_O emissions exhibited the same seasonal pattern whatever the treatment and the type of crop had little impact on the level of N_2_O emission [24]. In this study, N_2_O emissions were linearly associated with the N application rate. When considering the four consecutive crops studied here, seasonal N_2_O emissions had strong positive correlations with N application rates for each growing season (Fig 8A, S8 Fig) and the four cropping seasons (*r = 0*.8878**, *p<0*.*0001*, *n = 16*) (Fig 8B, S8 Fig). Besides, significant difference in cumulative N_2_O emissions was found among crop types, which mainly attributed to the influence of soil mineral N content and temperature factor. What’s more, the residual mineral nitrogen in the vegetable fields was also closely associated with the N application rate for each growing season (Fig 8C, S8 Fig) and four cropping seasons (*r* = 0.7745**, *p* = 0.0004, *n* = 16) (Fig 8D, S8 Fig).

### The influence of DCD and BC on N_2_O emissions

The application of nitrification inhibitors in agricultural soils is considered to be a promising approach for increasing N use efficiency and reducing N_2_O emissions to the environment [25]. The structure of DCD contains similar amino and imino functional groups in the NH_3_ structure, and this structure results in DCD in the form of substrate competition to disturb the use of ammonia oxidation on the substrate, thereby inhibiting the nitrification [26]. DCD had the most significant effect in reducing N_2_O emissions under the highest nitrogen application rate, and a higher rate of DCD will be more effective in reducing N_2_O emission [22, 27]. BC is widely used in soil improvement and allows for a reduction in carbon emissions because of its special functions and characteristics. The addition of BC into agricultural soils significantly increased soil total N, soil organic C and vegetable yield [28]. In this study, the results showed that both DCD and BC materials could effectively mitigate N_2_O emission fluxes and cumulative N_2_O emissions although the reduction mechanisms might be different for these two materials. The effects of DCD on reducing N_2_O emissions may attribute to the significant reduced the AOB amoA gene copy numbers especially with high nitrogen application rates [22]. However, the mechanism for N_2_O emission reduction of BC lies in how it affects many of the soil biogeochemical processes involved with the changes in organic carbon, nitrogen and enzymatic activities [29]. Unlike the DCD treatment, BC did not limit the availability of inorganic nitrogen to nitrifying and denitrifying bacteria; thus, the supply of ammonium and nitrate ions in the soil could not reveal inhibition of N_2_O emissions [30]. We also found no significant difference between the N2_BC treatment and conventional treatment in vegetable yields (data not shown). BC treatment significantly reduced accumulation of N_2_O emissions and yield-scaled N_2_O-N emissions, and this was beneficial for enhancing nitrogen use efficiency and reducing N loss caused by N_2_O release.

### The driving factors and evaluation indicator of N_2_O emissions

**Fig 9:**
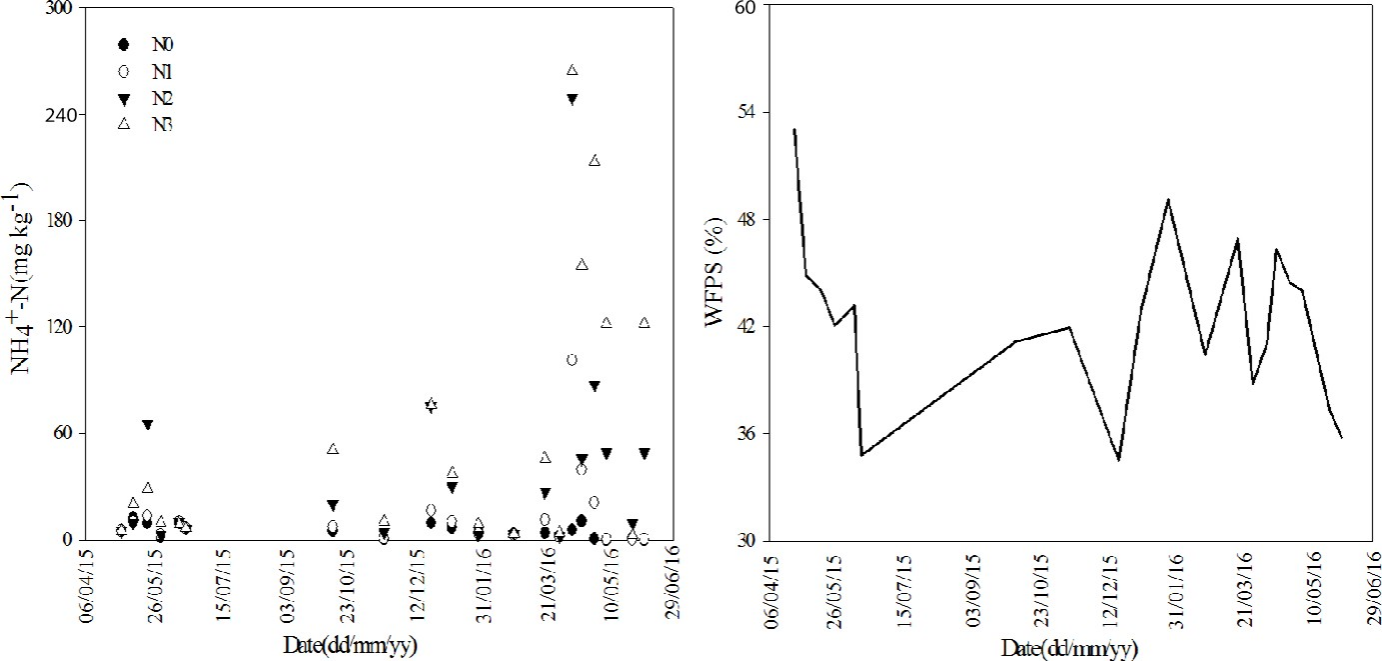
Dynamic changes of the content of soil NH4+-N and water-filled pore space (WFPS) during four vegetable growing periods. N0-no fertilizer N treatment; N1-low N application rate treatment (435 kg N ha^-1^); N2-conventional N application rate treatment (870 kg N ha^-1^); N3-high N application rate treatment (1305 kg N ha^-1^).

Soil moisture, air temperature and N application significantly affected N_2_O emissions [31–32]. In addition, the N_2_O emissions increase when soil pH decreases, and the addition of DCD resulted in a significant decrease in total N_2_O emissions in the acid condition and decreased peak N_2_O emissions in all pH treatments [33]. High content of soil available nitrogen, especially for ammonium nitrogen, caused higher N_2_O emissions of vegetables when compared with winter wheat fields [34–35]. The N_2_O emissions from soil with ammonium nitrogen fertilizer application were relatively higher than soil with nitrate nitrogen fertilizer application [36]. The present study also found an obvious correlation between peak N_2_O emissions and ammonium nitrogen content. The peak N_2_O emissions usually occurred within two weeks after the highest content of soil ammonium nitrogen (Fig 9, S9 Fig). The result indicated that an abundant accumulation of ammonium ions more likely resulted in an increase in loss of nitrous oxide gas from vegetable fields. Soil N_2_O emission flux and its source was closely related with the dynamic change of ammonium nitrogen and nitrate content in soil [37]. However, the WFPS in this study showed no significant correlation with N_2_O emissions.

In general, yield-scaled greenhouse gas (GHG) emissions provide a valuable measure for assessing the ability of management to mitigate emission without affecting by interaction management on crop productivity compared to area basis emission [38]. Therefore, the yield-scaled N_2_O emission can serve as an indicator to express N_2_O emissions in relation to crop productivity by calculating the N_2_O emissions per unit aboveground N uptake [18]. Yield-scaled N_2_O emissions changed greatly with the plantation crop species and different fertilization treatments. Besides, yield-scaled N_2_O emissions varied widely in agricultural soils, a level of variation that is caused by many factors, such as N source, climate, cropping system and sites [39–40]. Burzaco et al. [41] showed that yield-scaled N_2_O-N emissions increased with N application rates. In this study, the yield-scaled N_2_O emissions from the intensively fertilized vegetable fields were 8.2%–593.6%. These percentages were extremely higher than other reports and may have been partially caused by the high rates of precipitation, high temperatures, concentrated cultivated pattern and so on. The total N_2_O emissions from treatments in this study ranged from 32.3 to 89.7 kg N hm^-2^, accounting for 4.8% -7.4% of the total nitrogen input. The results of the present study indicated that emissions from vegetable fields are important potential sources of China’s N_2_O inventory. However, large uncertainties existed in the estimation of direct N_2_O emissions and background emissions of N_2_O from vegetable fields because different cropping systems have different emission characteristics, especially for these intensively managed vegetable fields. It also showed that yield-scaled N_2_O emissions were 22% lower with nitrapyrin than without the inhibitor at the same level of N fertilizer, but these did not interact with N rate or timing [41]. The result of this study also showed a significant reduction in yield-scaled N_2_O-N emissions with nitrification inhibitor and biochar treatment by 45.9% and 45.7% respectively compared with N2 treatment. The response of yield-scaled N_2_O-N emissions to fertilizer N addition was positive while to the addition of DCD and BC was negative, which mainly caused by the rate of increase in N_2_O emission comparison to aboveground N uptake. Therefore, minimizing yield-scaled N_2_O-N emissions could be realized by optimizing N application rates with high yields.

## Conclusions

In the present study, N_2_O emissions were linearly associated with the N application rate in vegetable fields. The N_2_O emissions occurred in pulses and the peak of N_2_O emissions varied greatly with crops and treatments. The peak value of N_2_O emissions increased with an increase in the N application rate. The total N_2_O emissions from treatments in this study ranged from 32.3 to 89.7 kg N hm^−2^, accounting for 4.8%–7.4% of the total nitrogen input. This finding indicated that emissions from vegetable fields are important potential sources of N_2_O emissions in China. Compared with the same amount of N fertilizer treatment, N2_DCD and N2_BC treatment significantly decreased cumulative N_2_O emissions by 34.6% and 40.8%, respectively. These results indicated that BC was better at reducing N_2_O emissions than DCD, particularly in the late growth stage of the four crops tested here. Yield-scaled N_2_O emissions varied greatly with crops under different N level treatments. Overall, this study provides insights for the effective technical measure related to inhibiting N_2_O emissions under field conditions in southern China. In addition, the yield-scaled N_2_O emissions also could be regarded as an environment parameter that can be used to evaluate N_2_O emission potential or calculate the N_2_O inventory. Furthermore, N management strategies also should be adjusted to enhance the efficiency of fertilizer use and provide for vegetable production without sacrificing yield and without the increasing N_2_O emissions. However, further study should be considered on the economic effects of controlling N_2_O emissions with the goal of providing environment friendly sustainable development.

## Supporting information

S1 Fig Original data for Fig 1. (XLSX)

S2 Fig Original data for Fig 2. (XLSX)

S3 Fig Original data for Fig 3. (XLSX)

S4 Fig Original data for Fig 4. (XLSX)

S5 Fig Original data for Fig 5. (XLSX)

S6 Fig Original data for Fig 6. (XLSX)

S7 Fig Original data for Fig 7. (XLSX)

S8 Fig Original data for Fig 8. (XLSX)

S9 Fig Original data for Fig 9. (XLSX)
